# Patterns in Decompression and Fusion Procedures for Patients With Lumbar Stenosis After Major Clinical Trial Results, 2016 to 2019

**DOI:** 10.1001/jamanetworkopen.2023.26357

**Published:** 2023-07-31

**Authors:** Rahul A. Sastry, Jia-Shu Chen, Belinda Shao, Robert J. Weil, Ki-Eun Chang, Ken Maynard, Sohail H. Syed, Patricia L. Zadnik Sullivan, Joaquin Q. Camara, Tianyi Niu, Prakash Sampath, Albert E. Telfeian, Adetokunbo A. Oyelese, Jared S. Fridley, Ziya L. Gokaslan

**Affiliations:** 1 Department of Neurosurgery, Warren Alpert Medical School, Brown University, Rhode Island Hospital, Providence, Rhode Island; 2 Department of Neurosurgery, Brain and Spine, Southcoast Health, Dartmouth, Massachusetts

## Abstract

**Importance:**

Use of lumbar fusion has increased substantially over the last 2 decades. For patients with lumbar stenosis and degenerative spondylolisthesis, 2 landmark prospective randomized clinical trials (RCTs) published in the *New England Journal of Medicine* in 2016 did not find clear evidence in favor of decompression with fusion over decompression alone in this population.

**Objective:**

To assess the national use of decompression with fusion vs decompression alone for the surgical treatment of lumbar stenosis and degenerative spondylolisthesis from 2016 to 2019.

**Design, Setting, and Participants:**

This retrospective cohort study included 121 745 hospitalized adult patients (aged ≥18 years) undergoing 1-level decompression alone or decompression with fusion for the management of lumbar stenosis and degenerative spondylolisthesis from January 1, 2016, to December 31, 2019. All data were obtained from the National Inpatient Sample (NIS). Analyses were conducted, reviewed, or updated on June 9, 2023.

**Main Outcome and Measure:**

The primary outcome of this study was the use of decompression with fusion vs decompression alone. For the secondary outcome, multivariable logistic regression analysis was used to evaluate factors associated with the decision to perform decompression with fusion vs decompression alone.

**Results:**

Among 121 745 eligible hospitalized patients (mean age, 65.2 years [95% CI, 65.0-65.4 years]; 96 645 of 117 640 [82.2%] non-Hispanic White) with lumbar stenosis and degenerative spondylolisthesis, 21 230 (17.4%) underwent decompression alone, and 100 515 (82.6%) underwent decompression with fusion. The proportion of patients undergoing decompression alone decreased from 2016 (7625 of 23 405 [32.6%]) to 2019 (3560 of 37 215 [9.6%]), whereas the proportion of patients undergoing decompression with fusion increased over the same period (from 15 780 of 23 405 [67.4%] in 2016 to 33 655 of 37 215 [90.4%] in 2019). In univariable analysis, patients undergoing decompression alone differed significantly from those undergoing decompression with fusion with regard to age (mean, 68.6 years [95% CI, 68.2-68.9 years] vs 64.5 years [95% CI, 64.3-64.7 years]; *P* < .001), insurance status (eg, Medicare: 13 725 of 21 205 [64.7%] vs 53 320 of 100 420 [53.1%]; *P* < .001), All Patient Refined Diagnosis Related Group risk of death (eg, minor risk: 16 900 [79.6%] vs 83 730 [83.3%]; *P* < .001), and hospital region of the country (eg, South: 7030 [33.1%] vs 38 905 [38.7%]; Midwest: 4470 [21.1%] vs 23 360 [23.2%]; *P* < .001 for both comparisons). In multivariable logistic regression analysis, older age (adjusted odds ratio [AOR], 0.96 per year; 95% CI, 0.95-0.96 per year), year after 2016 (AOR, 1.76 per year; 95% CI, 1.69-1.85 per year), self-pay insurance status (AOR, 0.59; 95% CI, 0.36-0.95), medium hospital size (AOR, 0.77; 95% CI, 0.67-0.89), large hospital size (AOR, 0.76; 95% CI, 0.67-0.86), and highest median income quartile by patient residence zip code (AOR, 0.79; 95% CI, 0.70-0.89) were associated with lower odds of undergoing decompression with fusion. Conversely, hospital region in the Midwest (AOR, 1.34; 95% CI, 1.14-1.57) or South (AOR, 1.32; 95% CI, 1.14-1.54) was associated with higher odds of undergoing decompression with fusion. Decompression with fusion vs decompression alone was associated with longer length of stay (mean, 2.96 days [95% CI, 2.92-3.01 days] vs 2.55 days [95% CI, 2.49-2.62 days]; *P* < .001), higher total admission costs (mean, $30 288 [95% CI, $29 386-$31 189] vs $16 190 [95% CI, $15 189-$17 191]; *P* < .001), and higher total admission charges (mean, $121 892 [95% CI, $119 566-$124 219] vs $82 197 [95% CI, $79 745-$84 648]; *P* < .001).

**Conclusions and Relevance:**

In this cohort study, despite 2 prospective RCTs that demonstrated the noninferiority of decompression alone compared with decompression with fusion, use of decompression with fusion relative to decompression alone increased from 2016 to 2019. A variety of patient- and hospital-level factors were associated with surgical procedure choice. These results suggest the findings of 2 major RCTs have not yet produced changes in surgical practice patterns and deserve renewed focus.

## Key Points

**Question:**

Did the use of lumbar decompression with fusion for the surgical treatment of lumbar stenosis with degenerative spondylolisthesis change after publication of 2 major randomized clinical trials in 2016 that demonstrated decompression with fusion was not superior to decompression alone?

**Findings:**

In this cohort study of 121 745 patients undergoing elective surgical procedures for diagnoses of lumbar stenosis with degenerative spondylolisthesis during inpatient admission, rates of decompression alone decreased from 32.6% in 2016 to 9.6% in 2019, and rates of decompression with fusion increased from 67.4% in 2016 to 90.4% in 2019.

**Meaning:**

These results suggest the findings of 2 major clinical trials have not yet produced changes in surgical practice patterns and warrant renewed focus.

## Teaser

This cohort study uses data from the National Inpatient Sample to assess changes in the national use of decompression with fusion vs decompression alone for the surgical treatment of adult patients with lumbar stenosis and degenerative spondylolisthesis in the US from 2016 to 2019.

## Introduction

In 2016, 2 prospective randomized clinical trials (RCTs)^1,2^ published in the *New England Journal of Medicine* sought to answer one of the most basic clinical questions in spinal surgical procedures: are patients with lumbar stenosis and degenerative spondylolisthesis optimally treated with decompression alone or in addition to lumbar fusion? Taken together, the results of these large studies,^1,2^ which were later extended by an even larger third RCT in 2021,^3^ suggest that addition of fusion confers only a modest decrease in long-term reoperation rates, without long-term functional benefit, compared with decompression alone among patients with lumbar stenosis and degenerative spondylolisthesis.^4^ Despite this high-quality evidence, recent updates to major spine society guidelines have been sparse.^5^ Furthermore, in the US, rates of decompression with fusion procedures for any number of indications have substantially increased over the last 20 years, even in excess of lumbar spinal pathology diagnoses in an aging population and despite the higher surgical risk profiles and aggregate and excess health care expenditures.^6,7,8,9,10,11,12,13^ The disproportionate growth in decompression with fusion procedures is likely dependent on multiple factors, including individual surgeon preferences, practice setting, surgeon experience, available implant technologies (including minimally invasive and anterolateral instrumentation options) and their aggressive marketing, and potential economic incentives.^7,13,14,15,16^ Given these recent patterns, it is unclear whether the results of these major studies^1,2^ have changed domestic surgical practice since their publication. We hypothesized that the results of these studies^1,2^ would result in a relative decrease in the use of decompression with fusion compared with decompression alone for patients undergoing surgical procedures for lumbar stenosis and degenerative spondylolisthesis from 2016 to 2019. As a secondary objective, we sought to assess which patient and hospital characteristics, if any, were associated with the decision to perform decompression with fusion compared with decompression alone in this patient population.

## Methods

### Data Source

The data used in this retrospective cohort study were obtained from the National Inpatient Sample (NIS), an administrative data set that captures a representative 20% of inpatient hospital encounters in the US.^17^ Although granular data regarding comorbidities or clinical outcomes may be lacking, the breadth of this particular data set makes it well suited to the study of longitudinal patterns across a variety of practice settings and reimbursement mechanisms. Given the transition of the NIS from *International Classification of Diseases, Ninth Revision, Clinical Modification* (*ICD-9*) to *International Classification of Diseases, Tenth Revision, Clinical Modification* (*ICD-10*) codes in 2015 and the lack of direct correspondence between relevant procedural codes in the 2 systems, only data from January 1, 2016, to December 31, 2019, were queried.^18,19,20^ All analyses were conducted, reviewed, or updated on June 9, 2023. Because this study used deidentified administrative data from a national database, it was deemed exempt from review and informed consent by the institutional review board of Rhode Island Hospital. This study followed the Strengthening the Reporting of Observational Studies in Epidemiology (STROBE) reporting guideline for cohort studies.^21^

### Participants

We first identified all adult patients (aged ≥18 years) in the NIS with inpatient hospital encounters for primary diagnoses corresponding to lumbar stenosis and lumbar degenerative spondylolisthesis using *ICD-10* diagnosis codes and procedure codes corresponding to lumbar or lumbosacral decompression and a single level, but not multilevel, lumbar or lumbosacral fusion. Patients were then categorized as undergoing decompression alone or decompression with fusion. This initial search yielded 191 510 admissions with 2780 corresponding *ICD-10* procedure codes.

**Figure 1.**
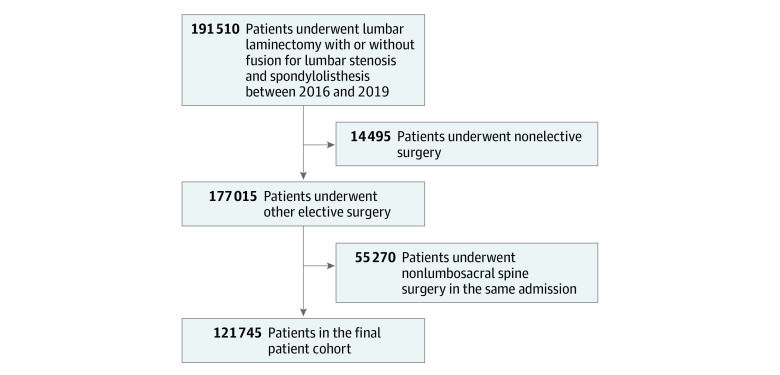
Patient Selection Diagram

Patients were excluded if they underwent nonelective surgical procedures, if their admissions were associated with other nondecompression or non–decompression with fusion *ICD-10* procedure codes, or if admissions were associated with any *ICD-10* procedure codes that implied either revision spine operations or management of nondegenerative pathologies, such as infection or tumor. In total, 121 745 patient admissions were included in the final analysis (Figure 1).

### Outcomes and Patient History Covariates

The primary outcome of this study was the use of decompression with fusion compared with decompression alone. Secondary outcomes included length of stay (LOS), total charges (adjusted for inflation to 2019 US dollars), total costs (adjusted for inflation to 2019 US dollars), mortality, and discharge disposition. Collected data included race and ethnicity categories as reported in the NIS (Asian or Pacific Islander, Black, Hispanic, Native American, White, and other; races and ethnicities included in the *other* category were not specifically identified), age (continuous), All Patient Refined Diagnosis Related Group (APR-DRG) illness severity (minor, moderate, major, or extreme), APR-DRG risk of death (referred to as *risk of mortality* in the APR-DRG database; minor, moderate, major, or extreme), hospital type (rural, urban teaching, or urban nonteaching), hospital region (Midwest, Northeast, South, or West), hospital size (small, medium, or large), and median income by patient residence zip code (reported as national quartiles, with quartile 1 indicating 25th percentile or lower, quartile 2 indicating 26th to 50th percentile, quartile 3 indicating 51st to 75th percentile, and quartile 4 indicating 76th percentile or higher). Race and ethnicity data were relevant to the study outcome because of substantial variation in both insurance coverage status and surgical decision-making across various surgical disciplines in the US. Sex did not differ significantly in any of the previous trials^1,2^ and was therefore not analyzed or reported in this study.

### Statistical Analysis

Patient demographic characteristics, hospital characteristics, and comorbidity indices were compared between the decompression alone and decompression with fusion cohorts using the Pearson χ^2^ test for categorical variables and the 2-tailed *t* test for continuous variables. To assess the associations between observed patient characteristics and choice of surgical procedure, multivariable logistic regression analysis using all eligible continuous and categorical variables was conducted. To avoid overestimation of significance and other sources of bias, variable selection methods and stepwise regression were not used.^22,23^ For all analyses, 2-tailed *P* ≤ .05 was considered significant. All data were analyzed using RStudio software, version 2023.03.1 (Posit).

## Results

Among 121 745 patients, the mean age was 65.2 years (95% CI, 65.0-65.4 years); most patients were of non-Hispanic White race and ethnicity (96 645 of 117 640 [82.2%]). A total of 21 230 patients (17.4%) underwent decompression alone, and 100 515 (82.6%) underwent decompression with fusion.

Longitudinal data regarding the use of decompression alone vs decompression with fusion for the management of lumbar stenosis and degenerative spondylolisthesis are presented in Table 1. The number of patients with lumbar stenosis and degenerative spondylolisthesis undergoing decompression with fusion increased from 15 780 of 23 405 (67.4%) in 2016 to 33 655 of 37 215 (90.4%) in 2019, representing a 113% increase over 3 years. In contrast, the number of patients with lumbar stenosis and degenerative spondylolisthesis undergoing decompression alone decreased from 7625 of 23 405 (32.6%) in 2016 to 3560 of 37 215 (9.6%) in 2019.

**Table 1.** Patients With Lumbar Stenosis and Degenerative Spondylolisthesis Undergoing Decompression Alone or Decompression With Fusion, 2016 to 2019

Type of surgical procedure	Patients, No. (%)			
	2016 (n = 23 405)	2017 (n = 28 335)	2018 (n = 32 790)	2019 (n = 37 215)
Decompression alone	7625 (32.6)	6475 (22.9)	3570 (10.9)	3560 (9.6)
Decompression with fusion	15 780 (67.4)	21 860 (77.1)	29 220 (89.1)	33 655 (90.4)

**Figure 2.**
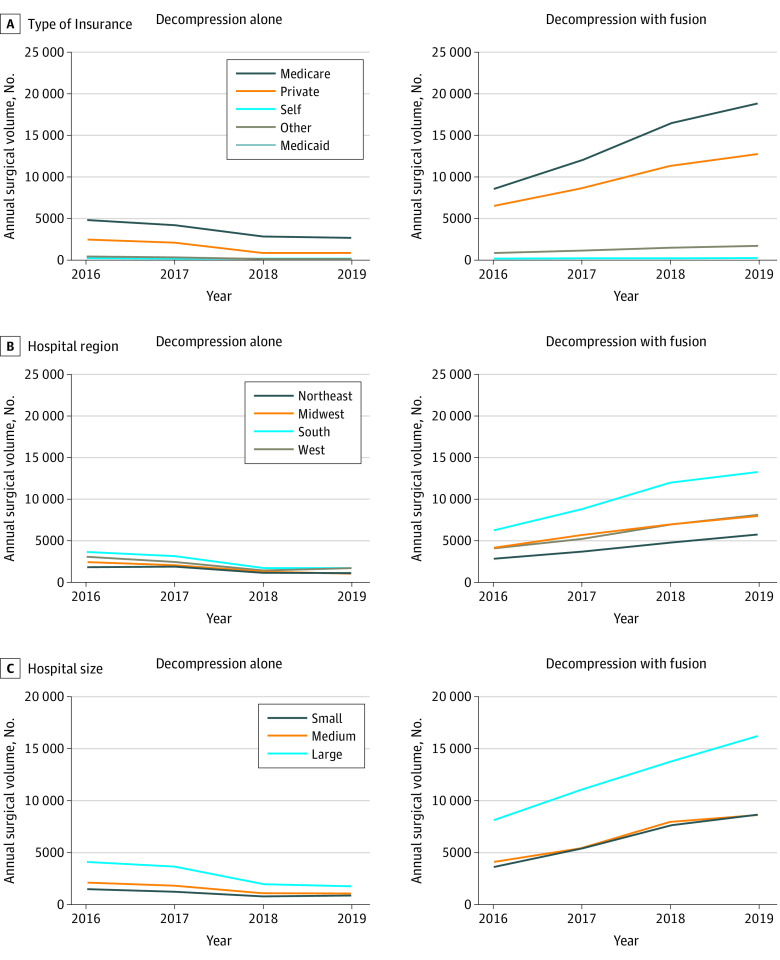
Surgical Volume Over Time for Decompression Alone and Decompression With Fusion Stratified by Insurance Type, Hospital Region, and Hospital Size

Demographic data for patients undergoing decompression alone vs decompression with fusion are presented in Table 2 and Figure 2. Compared with patients undergoing decompression alone, patients who underwent decompression with fusion were significantly younger (mean, 64.5 years [95% CI, 64.3-64.7 years] vs 68.6 years [95% CI, 68.2-68.9 years]; *P* < .001), more likely to have private insurance coverage (37 350 of 100 420 [37.2%] vs 5870 of 21 205 [27.7%]; *P* < .001), and less likely to have Medicare coverage (53 320 of 100 420 [53.1%] vs 13 725 of 21 205 [64.7%]; *P* < .001). Patients undergoing decompression with fusion vs decompression alone had significantly lower APR-DRG illness severity (eg, minor severity: 57 495 [57.2%] vs 11 590 [54.6%]; *P* = .02) and APR-DRG risk of death (eg, minor risk: 83 730 [83.3%] vs 16 900 [79.6%]; *P* < .001). Significant differences were also noted between the decompression with fusion vs decompression alone cohorts with regard to hospital region (eg, South: 38 905 [38.7%] vs 7030 [33.1%]; Midwest: 23 360 [23.2%] vs 4470 [21.1%]; *P* < .001 for both comparisons), hospital size (eg, small: 25 290 [25.2%] vs 4115 [19.4%]; *P* < .001), median income quartile by patient residence zip code (eg, quartile 4 [highest]: 25 790 of 99 005 [26.0%] vs 6450 of 20 875 [30.9%]; *P* < .001), and race and ethnicity (eg, non-Hispanic Black race and ethnicity: 7955 of 97 170 [8.2%] vs 1445 of 20 470 [7.1%]; *P* = .02). No significant differences were noted with regard to hospital type.

**Table 2.** Univariable and Multivariable Associations of Patient and Hospital Characteristics With Decision to Perform Decompression Alone vs Decompression With Fusion

Characteristic	Patients, No./total No. (%)			*P* value	Multivariable AOR of decompression with fusion (95% CI)
	Entire cohort (N = 121 745)	Decompression alone (n = 21 230)	Decompression with fusion (n = 100 515)		
Age, mean (95% CI), y	65.2 (65.0-65.4)	68.6 (68.2-68.9)	64.5 (64.3-64.7)	<.001	0.96 (0.95-0.96)
Year (relative to 2016)	NA	NA	NA	NA	1.76 (1.69-1.85)
Race and ethnicity					
Asian or Pacific Islander	2235/117 640 (1.9)	480/20 470 (2.3)	1755/97 170 (1.8)	.02	0.92 (0.72-1.17)
Black	9400/117 640 (8.0)	1445/20 470 (7.1)	7955/97 170 (8.2)		0.92 (0.80-1.06)
Hispanic	6240/117 640 (5.3)	1120/20 470 (5.5)	5120/97 170 (5.3)		0.86 (0.73-1.01)
Native American	545/117 640 (0.5)	95/20 470 (0.5)	450/97 170 (0.5)		1.19 (0.92-1.55)
White	96 645/117 640 (82.2)	16 955/20 470 (82.8)	79 690/97 170 (82.0)		1 [Reference]
Other^a^	2575/117 640 (2.2)	375/20 470 (1.8)	2200/97 170 (2.3)		0.92 (0.72-1.17)
Insurance type					
Medicare	67 045/121 625 (55.1)	13 725/21 205 (64.7)	53 320/100 420 (53.1)	<.001	1 [Reference]
Medicaid	5355/121 625 (4.4)	745/21 205 (3.5)	4610/100 420 (4.6)		0.83 (0.67-1.01)
Private	43 220/121 625 (35.5)	5870/21 205 (27.7)	37 350/100 420 (37.2)		0.98 (0.89-1.08)
Self-pay	670/121 625 (0.6)	140/21 205 (0.7)	530/100 420 (0.5)		0.59 (0.36-0.95)
Other	5335/121 625 (4.4)	725/21 205 (3.4)	4610/100 420 (4.6)		1.04 (0.85-1.26)
APR-DRG illness severity					
Minor	69 085/121 745 (56.7)	11 590/21 230 (54.6)	57 495/100 515 (57.2)	.02	1 [Reference]
Moderate	46 530/121 745 (38.2)	8550/21 230 (40.3)	37 980/100 515 (37.8)		1.07 (0.99-1.07)
Major	5705/121 745 (4.7)	1005/21 230 (4.7)	4700/100 515 (4.7)		1.20 (0.98-1.48)
Extreme	425/121 745 (0.3)	85/21 230 (0.4)	340/100 515 (0.3)		0.78 (0.36-1.68)
APR-DRG risk of death					
Minor	100 630/121 745 (82.7)	16 900/21 230 (79.6)	83 730/100 515 (83.3)	<.001	1 [Reference]
Moderate	17 755/121 745 (14.6)	3710/21 230 (17.5)	14 045/100 515 (14.0)		0.97 (0.87-1.08)
Major	2855/121 745 (2.3)	515/21 230 (2.4)	2340/100 515 (2.3)		1.10 (0.83-1.46)
Extreme	505/121 745 (0.4)	105/21 230 (0.5)	400/100 515 (0.4)		1.19 (0.59-2.44)
Hospital type					
Rural	4615/121 745 (3.8)	730/21 230 (3.4)	3885/100 515 (3.9)	.25	1 [Reference]
Urban nonteaching	28 075/121 745 (23.1)	5140/21 230 (24.2)	22 935/100 515 (22.8)		0.92 (0.69-1.23)
Urban teaching	89 055/121 745 (73.1)	15 360/21 230 (72.4)	73 695/100 515 (73.3)		0.94 (0.71-1.24)
Hospital region					
Northeast	19 345/121 745 (15.9)	3880/21 230 (18.3)	15 465/100 515 (15.4)	<.001	1 [Reference]
Midwest	27 830/121 745 (22.9)	4470/21 230 (21.1)	23 360/100 515 (23.2)		1.34 (1.14-1.57)
South	45 935/121 745 (37.7)	7030/21 230 (33.1)	38 905/100 515 (38.7)		1.32 (1.14-1.54)
West	28 635/121 745 (23.5)	5850/21 230 (27.6)	22 785/100 515 (22.7)		1.02 (0.88-1.18)
Hospital size					
Small	29 405/121 745 (24.2)	4115/21 230 (19.4)	25 290/100 515 (25.2)	<.001	1 [Reference]
Medium	31 705/121 745 (26.0)	5820/21 230 (27.4)	25 885/100 515 (25.8)		0.77 (0.67-0.89)
Large	60 635/121 745 (49.8)	11 295/21 230 (53.2)	49 340/100 515 (49.1)		0.76 (0.67-0.86)
Median income percentile of patient residence zip code					
≤25	25 370/119 880 (21.2)	3875/20 875 (18.6)	21 495/99 005 (21.7)	<.001	1 [Reference]
26-50	29 870/119 880 (24.9)	5115/20 875 (24.5)	24 755/99 005 (25.0)		0.90 (0.80-1.01)
51-75	32 400/119 880 (27.0)	5435/20 875 (26.0)	26 965/99 005 (27.2)		0.95 (0.84-1.06)
≥76	32 240/119 880 (26.9)	6450/20 875 (30.9)	25 790/99 005 (26.0)		0.79 (0.70-0.89)

Abbreviations: AOR, adjusted odds ratio; APR-DRG, All Patient Refined Diagnosis Related Group; NA, not applicable.

^a^ Races and ethnicities included in the *other* category were not specifically identified. All race and ethnicity categories were as reported in the National Inpatient Sample.

Results of multivariable logistic regression analysis for the primary outcome of decompression with fusion compared with decompression alone are also shown in Table 2. Factors associated with a significantly lower likelihood of undergoing decompression with fusion included older age (adjusted odds ratio [AOR], 0.96 per year; 95% CI, 0.95-0.96 per year), year after 2016 (AOR, 1.76 per year; 95% CI, 1.69-1.85 per year), self-pay insurance status (AOR, 0.59; 95% CI, 0.36-0.95), medium hospital size (AOR, 0.77; 95% CI, 0.67-0.89), large hospital size (AOR, 0.76; 95% CI, 0.67-0.86), and highest median income quartile by patient residence zip code (AOR, 0.79; 95% CI, 0.70-0.89). Factors associated with a significantly higher likelihood of undergoing decompression with fusion included hospital region in the Midwest (AOR, 1.34; 95% CI, 1.14-1.57) or South (AOR, 1.32; 95% CI, 1.14-1.54).

Secondary outcomes for patients undergoing decompression alone and decompression with fusion are summarized in Table 3. Overall, patients undergoing decompression alone vs decompression with fusion had shorter LOS (mean, 2.55 days [95% CI, 2.49-2.62 days] vs 2.96 days [95% CI, 2.92-3.01 days]; *P* < .001), lower adjusted total charges (mean, $82 197 [95% CI, $79 745-$84 648] vs $121 892 [95% CI, $119 566-$124 219]; *P* < .001), and lower adjusted total costs (mean, $16 190 [95% CI, $15 189-$17 191] vs $30 288 [95% CI, $29 386-$31 189]; *P* < .001). Patients undergoing decompression alone were also more likely to be discharged to an intermediate care or skilled nursing facility than those undergoing decompression with fusion (3415 of 21 230 [16.1%] vs 14 440 of 100 515 [14.4%]; *P* = .009). No significant differences were observed with regard to inpatient mortality.

**Table 3.** Outcomes of Patients With Lumbar Stenosis and Degenerative Spondylolisthesis Undergoing Decompression Alone vs Decompression With Fusion

Outcome	Patients, No. (%)			*P* value
	Entire cohort (N = 121 745)	Decompression alone (n = 21 230)	Decompression with fusion (n = 100 515)	
Length of stay, mean (95% CI), d	2.89 (2.85-2.93)	2.55 (2.49-2.62)	2.96 (2.92-3.01)	<.001
Adjusted total charges, mean (95% CI), $^a^	115 009 (112 923-117 095)	82 197 (79 745-84 648)	121 892 (119 566-124 219)	<.001
Adjusted total costs, mean (95% CI), $^a^	28 947 (28 101-29 793)	16 190 (15 189-17 191)	30 288 (29 386-31 189)	<.001
Mortality^b^	10 (0.01)	5 (0.02)	5 (0.005)	.23
Discharge disposition				
Home	79 825 (65.6)	13 865 (65.3)	65 960 (65.6)	.009
Short-term hospital	245 (0.2)	60 (0.3)	185 (0.2)	
ICF or SNF	17 855 (14.7)	3415 (16.1)	14 440 (14.4)	
Home health care	23 675 (19.4)	3865 (18.2)	19 810 (19.7)	

Abbreviations: ICF, intermediate care facility; SNF, skilled nursing facility.

^a^ Total charges and costs were adjusted for inflation to 2019 US dollars.

^b^ Percentages for mortality were based on 121 705 patients in the entire cohort (21 215 underwent decompression alone and 100 490 underwent decompression with fusion).

## Discussion

This cohort study found that, despite the simultaneous publication of 2 major RCTs^1,2^ demonstrating the long-term equivalence of decompression alone and decompression with fusion for the treatment of patients with lumbar stenosis and degenerative spondylolisthesis, the use of decompression with fusion continued to increase from 2016 to 2019 and comprised 90.4% of all inpatient surgical procedures undertaken for these pathologies in 2019. While the incidence of decompression with fusion and the increasing proportion of this procedure relative to decompression alone for the surgical treatment of lumbar stenosis and degenerative spondylolisthesis have increased over the past 2 decades in the US, this growth has occurred largely in the absence of high-quality clinical data to support its use collectively. Over the past decade, however, the optimal management of lumbar stenosis and degenerative spondylolisthesis has been studied carefully in 3 prospective RCTs.^1,2,3^

In the present study, multivariable logistic regression analysis, which accounted for multiple relevant confounders, found that patient insurance status, hospital location, and hospital size were all independently associated with the decision to perform decompression with fusion rather than decompression alone. Patients who underwent decompression with fusion over this period had longer LOS, higher total admission charges, and higher total admission costs than patients who underwent decompression alone.

The rapid growth of decompression with fusion procedures, which in our data set increased by 113% from 2016 to 2019, corroborates pre-2016 patterns (which we did not directly assess as a result of the transition from the *ICD-9* to the *ICD-10*) observed in a variety of data sources, including the NIS, Medicare claims database, Quality Outcomes Database, and American College of Surgeons National Surgical Quality Improvement Program, across a variety of degenerative lumbar spinal pathologies.^6,7,8,10,16,24,25,26,27,28^ Our results notably extended the results of Bae et al,^10^ who reported a fusion rate of 82.7% in patients with lumbar stenosis and degenerative spondylolisthesis (although this study included multilevel fusion procedures, which were excluded in the current study), and Al Jammal et al,^6^ who reported a fusion rate of 65.4% for all patients with lumbar stenosis regardless of lumbar spondylolisthesis codiagnosis. Although the transition from the *ICD-9* to the *ICD-10* made direct comparison of their associated epochs in the NIS challenging, the concordance of these values suggests that our observed patterns are likely explained by continued evolution in surgical practice and not recent variations in coding practice. Similarly, there has been a well-documented shift toward observation-status decompression and outpatient decompression procedures over the past decade, and decompression procedures captured in the NIS likely represent an older and frailer fraction of the overall population undergoing decompression procedures. It is noteworthy that the mean LOS for patients undergoing decompression alone in our analysis was only 0.41 days shorter than that of patients undergoing decompression with fusion (2.55 days vs 2.96 days); in contrast, in a meta-analysis of major studies regarding difference in LOS,^12^ the estimated difference was approximately 1.7 days. While definitive national data are lacking, the rate of outpatient decompression among all decompression procedures is likely 50% to 60%^29,30^; furthermore, there is no evidence that the relative proportion of outpatient decompression procedures has been increasing over time.^29,30^ The patterns identified in our analysis were not subtle or ambiguous. To offset the 3-year growth in decompression with fusion procedures, one would need to suggest that nearly 13 000 additional decompression procedures, almost 4 times the observed number in 2019, both occurred and were shifted to outpatient settings.

The continued increase in the use of decompression with fusion procedures in the US was unexpected and meaningful in the context of observed patterns among health care systems in other countries, such as those of Finland and Denmark, in which rates of decompression with fusion have either plateaued or declined substantially since 2016.^31,32^ One of many possible explanations for this observation is that the findings from relevant RCTs,^1,2,3^ 2 of which were conducted in Europe,^2,3^ are not applicable to clinical practice, especially in a US patient population. Some of the challenges noted by US spine surgeons in response to published trial results include ambiguity about the definition of spondylolisthesis and omission of contemporary definitions of spinopelvic deformity parameters,^33,34^ baseline comorbidity differences between European and US patients,^35^ variation in the types of fusion or decompression performed,^36,37^ significance of high reoperation rates for decompression alone,^36^ and intrinsic differences in reoperation decisions between European and US spine surgeons.^36^ While the stated desire to identify subpopulations of patients with lumbar stenosis and degenerative spondylolisthesis who are optimal candidates for decompression with fusion is likely both correct and well intentioned,^34,35^ it is also incompatible with the observed use of decompression with fusion in excess of 90% in the current US patient cohort.

Translation of relevant and accepted high-quality clinical evidence and deimplementation of low-value surgical practice is also difficult. Barriers include, but are not limited to, financial incentives, lack of guidelines and/or national regulations, and delayed evidence dissemination through medical education or professional societies.^38,39^ In our analysis of the use of decompression with fusion for the treatment of lumbar stenosis and degenerative spondylolisthesis, we found that self-pay insurance status, hospital region, and hospital size were associated with a significantly higher likelihood of undergoing decompression alone vs decompression with fusion. The use of decompression with fusion was associated with substantially higher total hospital charges and costs. Other analyses from the Quality Outcomes Database and the NIS have previously implicated hospital teaching status, surgical experience, private or for-profit hospital status, and insurance status as explanatory factors in the decision to perform decompression with fusion.^14,27,40^ It has been relatively well established that, in various surgical contexts including spine procedures, fee-for-service reimbursement schemes incentivize maximal surgical interventions and therefore play a role in variations in surgical practice that are not justifiable in the context of available medical evidence.^39,41,42,43^ Together, these results strongly suggest procedural reimbursements (or lack thereof, as is implied by the recent stasis in Medicare reimbursements for decompression with fusion procedures relative to inflation^44^) and a variety of non–medical-related considerations may be associated with the move toward fusion procedures even as high-quality RCT data are increasingly available.^7,13,16^ Furthermore, few spine practice guidelines were updated in light of the simultaneous 2016 publication of the first 2 major RCTs.^1,2,5^ In addition, the value of guidelines in practice modification should also be interrogated; a recent survey^15^ by the North American Spine Society did not find significant differences in lumbar spine practice patterns between surgeons who reported following guidelines in their decision-making and those who did not.

### Strengths and Limitations

This study has strengths. As previously noted, the NIS captures a representative sample of all US hospitalizations with a high degree of data fidelity. It is therefore appropriately suited to questions regarding longitudinal data patterns.

The study also has limitations. Because the NIS only includes data on inpatient encounters, it does not capture surgical procedures conducted in outpatient settings or patients who were otherwise not categorized as inpatients. Furthermore, NIS procedures are identified with *ICD* codes rather than the comparatively more useful *Common Procedural Terminology* system. To mitigate this ambiguity, we used a 2-step review of included *ICD* codes to exclude patients undergoing surgical procedures for nonspine or nondegenerative pathologies; it is unclear to what extent, if any, this method was used in previous NIS studies on the topic (and, notably, the essential study results are robust to these manipulations, although these data are not formally reported in this article). It is possible that recorded diagnosis codes may not reflect the true complexity of conditions or symptoms among patients undergoing surgical procedures^45^; nevertheless, the observed patterns from more than 100 000 hospital admissions provide important clarity based on the large numbers, the patterns over time, and the geographic variability provided by the NIS. Future analyses should evaluate these patterns after 2019 and 2021, when the NORDSTEN-DS (Norwegian Degenerative Spondylolisthesis and Spinal Stenosis) trial results were published,^3^ as data become available.

## Conclusions

In this cohort study using a representative national data set, the rate of decompression with fusion procedures for the management of lumbar stenosis and degenerative spondylolisthesis increased substantially in the 3-year period after the publication of 2 major RCTs^1,2^ that showed decompression with fusion was not superior to decompression alone for the surgical treatment of lumbar stenosis and degenerative spondylolisthesis. When adjusting for available confounding factors, this study found that patient insurance status, age, and hospital characteristics were all associated with the decision to perform decompression with fusion compared with decompression alone. These results suggest that the findings of 2 major RCTs^1,2^ in this area of spinal procedures have not yet changed practice patterns and deserve renewed focus.

## Notes

Supplement.
Data Sharing Statement
