# Supplementary material for: Patterns in Decompression and Fusion Procedures for Patients With Lumbar Stenosis After Major Clinical Trial Results, 2016 to 2019
Source: JAMA Netw Open. 2023 Jul 31;6(7):e2326357. doi: 10.1001/jamanetworkopen.2023.26357 (PMC10391306; doi:10.1001/jamanetworkopen.2023.26357)
Supplement: Supplement. — Data Sharing Statement [file jamanetwopen-e2326357-s001.pdf]

Data Sharing Statement

Sastry. Patterns in Decompression and Fusion Procedures for Patients With Lumbar Stenosis After Major Clinical Trial Results, 2016 to 2019. *JAMA Netw Open*. Published July 31, 2023. doi:10.1001/jamanetworkopen.2023.26357

Data
Data available: No
